# Obesity and Maternal-Placental-Fetal Immunology and Health

**DOI:** 10.3389/fped.2022.859885

**Published:** 2022-04-28

**Authors:** Meredith Monaco-Brown, David A. Lawrence

**Affiliations:** ^1^Department of Pediatrics, Bernard and Millie Duker Children’s Hospital at Albany Medical Center, Albany, NY, United States; ^2^New York State Department of Health, Wadsworth Center, Albany, NY, United States; ^3^Department of Environmental Health Sciences, University at Albany School of Public Health, Rensselaer, NY, United States

**Keywords:** maternal obesity, placenta, inflammation, oxidative stress, trophoblasts, Th cells, macrophages

## Abstract

Obesity rates in women of childbearing age is now at 29%, according to recent CDC reports. It is known that obesity is associated with oxidative stress and inflammation, including disruptions in cellular function and cytokine levels. In pregnant women who are obese, associated placental dysfunction can lead to small for gestational age (SGA) infants. More frequently, however, maternal obesity is associated with large for gestational age (LGA) newborns, who also have higher incidence of metabolic disease and asthma due to elevated levels of inflammation. In addition, anthropogenic environmental exposures to “endocrine disrupting” and “forever” chemicals affect obesity, as well as maternal physiology, the placenta, and fetal development. Placental function is intimately associated with the control of inflammation during pregnancy. There is a large amount of literature examining the relationship of placental immunology, both cellular and humoral, with pregnancy and neonatal outcomes. Cells such as placental macrophages and NK cells have been implicated in spontaneous miscarriage, preeclampsia, preterm birth, perinatal neuroinflammation, and other post-natal conditions. Differing levels of placental cytokines and molecular inflammatory mediators also have known associations with preeclampsia and developmental outcomes. In this review, we will specifically examine the literature regarding maternal, placental, and fetal immunology and how it is altered by maternal obesity and environmental chemicals. We will additionally describe the relationship between placental immune function and clinical outcomes, including neonatal conditions, autoimmune disease, allergies, immunodeficiency, metabolic and endocrine conditions, neurodevelopment, and psychiatric disorders.

## Introduction

### Obesity Prevalence and Significance

Obesity is a medical crisis with increasing rates both in the United States (1–5) and worldwide (5–7). Based on CDC and WHO definitions, normal weight ranges from a BMI of 18.5 to 24.9, and overweight is defined as BMI ≥ 25. Obesity is defined as BMI ≥ 30, and severe obesity is defined in different sources as ≥35 or 40. In the United States general population, adult obesity rates have increased from 30.5 to 42.4% from the year 2000 to 2018 (2), with global rates also increasing significantly, from 7% in 1980 to 12.5% in 2015, with similar trends upward despite a large range of prevalence based on regions, demographics, and socioeconomic differences (5). Obesity-related conditions account for as much as 20% of healthcare spending in the United States totaling $190 billion annually (8), with proportionately high amounts spent world-wide (9).

Obesity rates in women of childbearing age is 31.8%, with half of that group in the severe obesity range (10). Obesity-related conditions in women include diabetes, hypertension, PCOS, as well as many other conditions which create significant risks to fertility and conception (11), and to pregnancy and maternal health before, during, and after delivery. These conditions also have been shown to have significant effects to offspring health, both in the perinatal period (12) and in later childhood and adulthood. The effect of maternal obesity on the adult phenotype of offspring is a common example of Barker’s hypothesis of fetal origins of adult disease (13).

Obesity does not affect all races or socioeconomic groups similarly. Obesity rates in women differ widely based on race, with Non-Hispanic Black women at a prevalence of 56.1% vs. Hispanic women at 48.4%, Non-Hispanic White women at 38.8%, and Non-Hispanic Asian women at 13.6%. Higher education level decreases risk for obesity, as does former or current smoking history. In addition, obesity rates increase in women in less urbanized areas (14). These different factors may affect the development and perpetuation of obesity in different ways including access to nutritious food, access to activity, and various cultural and regional practices. Many of these associated demographic factors are also relevant to other sources of maternal stress, such as infections, environmental toxicants, and psychosocial stressors. Additionally, there are transgenerational influences that may be influencing obesity rates that coincide with the changes in diets, increasing exposures to environmental pollutants, and the concomitant effects of climate change.

Because obesity is so prevalent in women of childbearing age and has so many concerning effects on the mother and her offspring, and because this epidemic is differentially affecting women in marginalized populations, it is critical that we understand the mechanisms of these effects in order to be able to target preventative and therapeutic strategies that may improve outcomes for all communities. As obesity can affect maternal-placental-fetal health and the developmental origins of offspring immunity (15, 16), which can influence lifetime health, the converse concept of the offspring’s immune system increasing obesity incidence is also suggested (17). It’s important to note that with increasing obesity there has been more incidence of immunopathologies such as asthma, allergies, autism and some autoimmune diseases as well as enhanced susceptibility to infections (18) and cancers (19).

### Maternal Stressors in Pregnancy

There is increasing evidence that multiple forms of environmental stress during the prenatal period can induce a lifetime of adverse health effects. Regarding the fetus and offspring, the exogenous and endogenous effects on the mother include diet, which can be influential as discussed in papers about the developmental origins of adult diseases. In fact, the influences of malnutrition or a fat rich diet may be transgenerational (20). A rich diet can lead to maternal obesity, which directs paths to metabolic dysfunction and inflammation, and maternal adiposity also increases fat deposition in the placenta and fetus, which affect the developing types of fetal immune cells (21). It is generally believed that these early developmental stresses affect the offspring due to epigenetic and metabolic changes (22).

### Maternal and Fetal Cells at Interface and Beyond

The placenta plays a vital role in fetal development. The placenta is unique in that its cell and molecular composition of maternal and fetal tissues influence the maternal delivery of nutrients as well as hormones, cytokines, antibodies, and cells to the fetus, helps to protect mother and fetus during this semi-allogenic relationship (23), and is rejected to enable parturition. In addition to the molecular effects on the fetus, fetal microchimerisms (FMCs) are established during and after pregnancy with beneficial (24) or adverse (25) health consequences for mothers and offspring. These positive and negative effects involve maternal immunity. Conversely, maternal microchimerisms (MMC) may detrimentally affect some offspring. Two rare detrimental outcomes are neonatal lupus (26) and type 1 diabetes (27), which are autoimmune diseases resulting in part from maternal cells in offspring.

**FIGURE 1:**
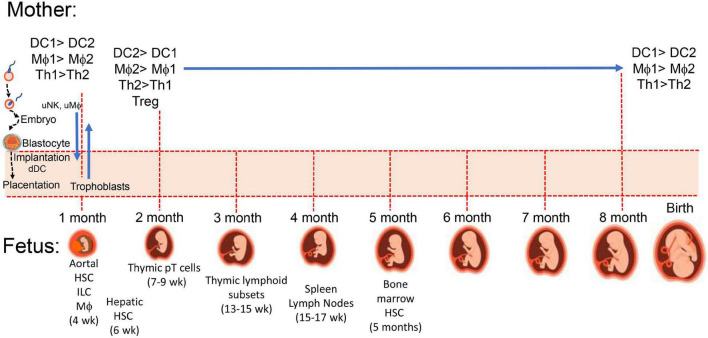
Maternal-placental-fetal immune systems. During implantation and placentation embryonic stem cells and mature maternal immune cells interact to establish maternal immune tolerance to semi-allogenic trophoblasts which invade maternal uterus along with fetal hematopoietic stem cells (HSC) developing from the paraaortic splanchnopleure (aortal) tissue (188). At 4 weeks post-conception, there are myeloid-derived microglia cells in the developing brain, which influence development of neuronal connections. Fetal thymus starts accumulating myeloid cells to support increasing presence of precursor lymphoid T (pT) cells (189) for later development of CD4 and CD8 single positive and γδ TCR^+^ thymocytes, and CD4^+^CD25^+^ T regulatory cells (189). Proportions of macrophages and neutrophils (171) as well as B cells shift from liver to marrow between 5 and 16 weeks.

Maternal immunity plays a critical role in pregnancy and the development of healthy offspring. Immune cells aid (i) implantation of the trophoblasts into the uterine decidua and the peripheral maternal system, (ii) placental development, (iii) angiogenesis (28–30) to establish needed delivery of nutrients and maternal factors, and (iv) parturition as outlined in Figure 1. While maintaining host defense against pathogens, maternal immune cells assist or initiate implantation, placentation, and parturition at the appropriate time, and in the intervening period, maternal immunity can help or hinder fetal development (31). Maternal immunity can help by preventing fetal access of pathogens and transferring protective antibodies to the fetus and hinder by delivery of proinflammatory cytokines, antibodies to fetal antigens, and inappropriate levels of steroids. There are additional aspects of the maternal systemic environment affecting fetal development that are mentioned throughout this review involving neuroendocrine and immune network interactions. For example, maternal obesity can affect the number of maternal macrophages in the placenta and enhance numbers of innate immune cells promoting inflammation, oxidative stress, and mitochondrial and metabolic dysfunction (32–35). Obesity is adipose tissue overload in organs, including the placenta (36), and it influences metabolic complications associated with mitochondrial dysfunction (37). Cardiac dysfunction related to obesity (38) may be especially problematic during pregnancy with the extra vascular remodeling needed for the fetus and increased circulating maternal blood volume. Inadequate or inappropriate delivery of nutrients, cells and cellular products to the fetus could lead to preterm birth and/or underweight births. Placental dysfunction contributes to spontaneous preterm births (SPTBs) and is related to placenta metabolism affected by mitochondria dysfunction and inflammation, which was reported to display sex disparity with more transcriptomic differences with male SPTB placentas (39). The placentas of male fetuses have been reported to have a profile more inflammatory than the placentas of females, which have more control of immunity and regulation of endocrine involvement and placental growth (40, 41).

### Environmental Stresses

Maternal stress may be induced by an infection, environmental pollutants, or physical or psychologic disturbances. Each stressor alone or combined with another may have profound influence on maternal-placental-fetal immunology and the developing fetus, which could affect offspring health for a lifespan (42, 43). Maternal depression and obesity combine to affect fetal development and the offspring’s mental and physical health (44). An emotional stress or pollutant exposure during pregnancy is a risk factor for offspring with increased potential development of later cardiovascular disease, cancer, or autoimmune disease (45–48). Perinatal maternal stresses also can impact the offspring’s neurodevelopment (49–52).

Maternal obesity is associated with maternal, placental and fetal metabolic dysfunction with enhanced inflammation (21, 53). “Metaflammation” was the term coined by Gregor and Hotamisligil (54) for the chronic, low-grade inflammatory state associated with obesity, which differs from acute inflammatory responses induced by pathogen associated molecular patterns (PAMPs) or damage associated molecular patterns (DAMPs). Metaflammation is triggered by metabolites and nutrients and may lead to systemic insulin resistance due to inflammatory mechanisms associated with obesity (54, 55). Early inflammation affects the developing immunophenotypes of fetal immune cells, which likely relate to obesity effects on epigenetics and the microbiome (56, 57).

### Endogenous Stresses

Macrophages in adipose tissue can polarize and affect bioenergetics with obesity (58). With more adiposity, there is more inflammation in tissues due to more fat creating oxidative stress-induced cell damage and release of DAMPs to stimulate pattern recognition receptors (PPRs) such as toll-like receptors (TLRs), which induce proinflammatory cytokines and chemoattractants (chemokines) influencing influx of macrophages, which includes into the placenta. Trophoblasts attract endometrial stomal cells (59) and placentas attract many different immune cells (60). The influence of maternal obesity on fetal inflammation has been reported to be mainly due to regulatory effects on the placenta (61), which may be due to a low circulating level of adiponectin (62). Stress prior to or during pregnancy affects placental development due to posited dysregulated neuroendocrine immune interactions, which cause long-term alterations to the immune and nervous systems of offspring. Psychological stress affects the development of the placenta, which includes placental gene expression and oxidative stress (63); oxidative stress leads to placental pathology and detriments to fetal development (64, 65). Normally, the placenta helps skew the maternal and fetal environment toward a CD4^+^ helper T cell type-2 (Th2) and anti-inflammatory profile (66); however, as mentioned earlier, obesity and other stresses create a more CD4^+^ helper T cell type-1 (Th1) and inflammatory gestational environment. Additionally, in normotensive pregnancies, the placenta helps to control the level of stress hormones trafficking to fetus (67); the placenta attempts to control the multiple forms of maternal stress on the fetus (68).

Preeclampsia may begin as early as placentation which is when fetal trophoblasts and maternal uterine cells are aided by uterine immune cells to achieve efficient implantation for proper vascularization (69, 70). Inadequate vascularization will affect placental and fetal growth and is associated with preeclampsia. Stress in the placenta was observed with placental expression of soluble fms-like tyrosine kinase-1 (sFlt-1) and triglycerides in maternal serum (71) and is often accompanied with maternal hypertension and proteinuria, which is induced by sFlt-1 (sVEGFR1); sFlt-1 is an anti-angiogenic protein, because it interferes with vascular endothelial growth factor (VEGF), which triggers angiogenesis. VEGF also has been suggested to recruit macrophages (Mφs) and aid shift toward type-2 Mφs (Mφ2), which enhances immune tolerance and tissue remodeling (72). The endothelial dysfunction in the placenta increases the likelihood of preeclampsia along with an immunophenotype skewing more toward Th1 cells producing proinflammatory cytokines (73). However, clinical signs of preeclampsia usually don’t become apparent until the beginning of the 2nd trimester. Early signs of preeclampsia may come from metabolomics (74). Since preeclampsia has higher prevalence with maternal obesity, metabolites predictive of oxidative stress might be informative. One such metabolite is acylcarnitine, a product of fetal fatty acid oxidation disorders (75). An accumulation of acylcarnitine may be indicative of mitochondrial dysfunction or peroxisome to mitochondria processing (76). Mitochondria are posited to be the intermediary between obesity and preeclampsia since higher levels of fatty acids can lead to more reactive oxygen species (ROS) generated by mitochondria in tissues including the placenta (77). Oxidative phosphorylation by mitochondria leads to production of ATP and ROS needed for maternal-placental-fetal cellular functions. Early in pregnancy ROS triggers expression of VEGF and glucose transporters to promote angiogenesis (78); however, too much ROS leads to mitochondrial dysfunction causing placental inflammation and epigenetic changes to fetus that can affect offspring health for life (79, 80). In a rat model of ROS-mediated oxidative stress caused by hyperandrogenism and insulin resistance, fetal loss was associated with dysregulation of the placental mitochondria–ROS–SOD1/Nrf2 axis (81).

Prenatal maternal stress, which includes maternal obesity, affects fetal growth by regulating production of metabolites as mentioned earlier and by influencing delivery of maternal products such as glucocorticoids and nutrients. Glucocorticoids are essential for fetal development and their level is under maternal hypothalamic-pituitary-adrenal (HPA) axis control. Starting in the 2nd gestational trimester, the placenta secretes corticotrophin-releasing hormone to promote cortisol release (82). Obese pregnant women have low cortisol levels throughout pregnancy (83). Obese pregnant women also have a blunted HPA axis, and it has been suggested that maternal obesity increases 11β-hydroxysteroid dehydrogenase-2 (11β-HSD-2) activity, which metabolizes cortisol to inactive metabolites so that glucocorticoid receptor (GR) is not signaled (84). Conversely, maternal depression may lower placental expression of 11β-HSD-2 allowing too much access of glucocorticoid to the fetus. Both over and under delivery of glucocorticoids to the fetus can be detrimental. Glucocorticoids directly affect the fetus and placental production of neurosteroids and neurohormones, which includes regulation of the HPA axis (85–87). Stress also affects nutrient delivery to the fetus (88), and O-linked-N-acetylglucosamine transferase (OGT), a placental nutrient sensor, is involved with placental epigenetics. OGT affects long-term neurodevelopmental programming, which includes programming of the HPA axis (89, 90). Together, these stress-related modulations enhance long-term detrimental effects on offspring, which includes increased prevalence of metabolic and cardiovascular disorders, and neurodevelopmental sequelae. However, exactly how stress mediates these detrimental outcomes is unclear and stress from obesity may involve different pathways than that from other forms of stress. Placental expression of 11β-hydroxysteroid dehydrogenase type 2 (11β-HSD2) is responsible for preventing high maternal glucocorticoid levels from affecting the placenta and fetus (91). The expression of 11β-HSD2 increases through term in the human placenta (92). Analysis of human mothers with mental health problems have been reported to have lower 11β-HSD2 expression (93), which could propagate mental health issues in offspring, and neurodevelopment is modulated by placental stress effects (94). Interestingly, the steroidogenic pathway for glucocorticoids is shared with progesterone, and an imbalance between progesterone and glucocorticoid has been suggested to cause placental insufficiency, inflammation, and maternal immunity unfriendly toward the fetus (95). Although cortisol is the HPA product often associated with detrimental fetal effects from maternal stress, many other factors have been implicated such as catecholamines, cytokines, serotonin/tryptophan, ROS and maternal microbiota (96).

### Maternal-Placental-Fetal Immune Cells During Pregnancy

**FIGURE 2:**
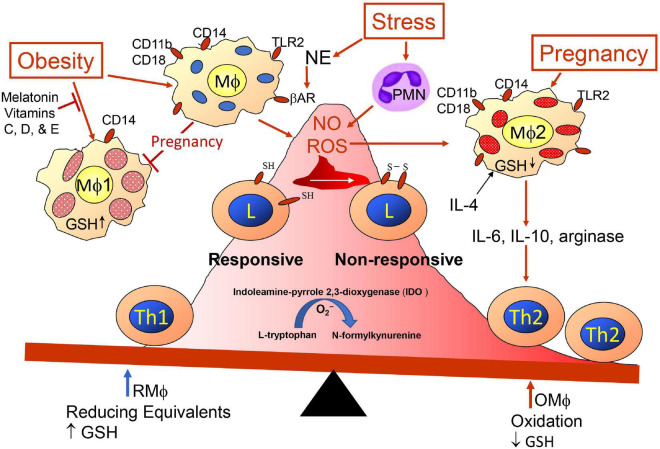
Redox imbalance promoted by obesity, pregnancy and stress. Maternal immunity is skewed more toward Th2 immunity (anti-inflammation) regarding presence of semi-allogenic fetus developing within uterine tissue. Balance is influenced by pregnancy, obesity, and exposome, which may have additive, synergistic, antagonistic or potentiating affects. Redox status is influenced by glutathione (GSH) availability. Psychological stress modifies immunity through sympathetic nerve release of norepinephrine (NE) triggering beta-adrenergic receptors (β-AR) on Mφs (190) and release of polymorphonuclear cells (PMN) from bone marrow (191). Toll-like receptors (TLRs) are triggered by PAMPs and DAMPs.

The mother’s innate and adaptive immune cells play a key role in all phases of the pregnancy, and as mentioned earlier, maternal immunity needs to respond appropriately for embryo implantation, placentation allowing semi-allogenic cells into maternal tissue with activation of immunotolerance to the paternal antigens while maintenance of immunity to other foreign antigens, and finally disruption of the cohabitation for parturition. Hormone and cytokine/chemokine levels vary at different stages of the pregnancy; they initially aid immune disruption of the epithelial uterine barrier for decidualization, they help to maintain local unresponsiveness to the fetal antigens until at parturition, and finally they again convert to inflammatory processes to aid placental release (97–99). Maternal decidual innate immune cells assist early invasion of the uterus by fetal trophoblasts, and Mφ-derived angiogenic factors promote development of vascularization establishing utero-placental circulation. This alone indicates the dynamics of the immune cells because the invasion requires destruction of the epithelial barrier allowing penetration of the semi-allogenic blastocyst through this barrier and entrance into the uterine musculature/tissue (100). To break through the epithelial barrier would require activities of innate natural killer (NK) cells and Mφs promoting inflammation and breakage of epithelial tight junctions with assistance from many other maternal cells such as dendritic cells (DCs), mast cells releasing matrix metalloproteinases (MMPs) and T cells (101), but this would have to be accomplished with no damage to the embryo so entrance relates to the activities of the trophoblasts cooperating with maternal immune cells. However, any weakness in the uterine wall barrier could also allow entry of bacteria, so immune defenses would be needed by mature type-1 Mφs (Mφ1) and DC (DC1) and Th1 cells; therefore, there needs to be plasticity of Mφs, DCs, and lymphoid subsets (102, 103). For implantation and placentation, unlike DC1 and Mφ1, there is an abundance of immature DC cells expressing CD209 (DC-SIGN^+^) cells (104) and Mφ2 producing immunosuppressive tumor growth factor-beta (TGF-β), IL-10, and indoleamine 2,3-dioxygenase (IDO) (105). DC-SIGN^+^ cells are required for expansion of CD4^+^Helios^–^Foxp3^+^ adaptive Treg (iTreg) cells and together help to maintain tolerance to placental and fetal antigens until near time for birth, and earlier loss of DC-SIGN^+^ and iTreg cells will aid preeclampsia (106). Mφ1 and DC1 preferentially activate Th1 cells and Mφ2 and DC2 activate Th2 cells; Th1 response creates more oxidative stress and the Th2 response attempts to mitigate the stress (107, 108). The Mφ1 and Mφ2 balance is affected by oxidative stress on Mφs and/or Th cells (Figure 2). The oxidative stress might be lessened with better diet including an increase in vitamins (78, 109–114). Melatonin (78, 115–119) also may mitigate ROS effects and as well as affect sleep. Balance of Mφ1 and Mφ2 is important for a normal pregnancy, but the ratio may vary at different stages (28, 120, 121). When preeclampsia develops Mφ1 predominate (122–124). The Mφ1 and an environment with their products such as IL-1β, TNFα, MMPs, and nitric oxide (NO) are effective in terminating a normal pregnancy but can initiate preterm labor (125). Some trophoblasts may undergo some damage during implantation, but the maternal immune system should remain unresponsive or tolerant to the implanted developing blastocyst and should aid angiogenesis, which would be similar to wound healing as assisted by Mφ2 and decidual natural killer cells (dNK), which are more growth promoting and angiogenic than cytotoxic (100). In the 1st trimester, the predominant dNK are dNK1 (∼55%), which may aid immunotolerance to extravillous trophoblasts (EVTs) and dNK2 (∼15%), which produce more interferon-gamma (IFNγ) and may aid implantation (126). The EVTs, dNK subsets, and decidual macrophages (dMφ) seem to work as a team in remodeling the spiral artery. So there also is need for establishment of tolerance to the paternal antigens and wound healing for development of the vascular placenta. The growth promoting dNK are reported to be CD49a^+^PBX homeobox 1 (PBX1)^+^ Eomes^+^ (127) and produce pleiotrophin, osteoglycin, and osteopontin (128). Absence or mutated PBX1 (PBX1^*G*21*S*^) affects fetal growth and increases prevalence of spontaneous abortion (127). Three uterine Mφ subsets have been immunophenotypically defined CCR2^–^CD11c^*LO*^ (CD11c^*low*^, ∼80%), CCR2^–^CD11c^*HI*^ (CD11c^*high*^, ∼5%), and CCR2^+^CD11c*^HI^* (CD11c*^high^*, 10–15%) in the 1st trimester (129). The dMφ subset(s) may be a unique linkage unlike that of the bone marrow stem cell derived Mφ1 and Mφ2 subsets (130). Like Mφs, DCs, which are more efficient in presenting antigen for activation of T cells, are in decidua tissue, but at a lower number than Mφs. DCs also show plasticity of phenotype and function (131) including maintenance of immune tolerance (132), which has been suggested to be important for immune control during pregnancy (133). Pregnancy complications may develop when there is a decline in dMφ expressing CD163, CD206, and CD209, which secrete the immunosuppressive factors IL-10, TGF-β, and IDO. This is accompanied by a concomitant increase of dMφ expressing CD80, CD86, and MHCII, which along with Th1 release TNFα, IFNγ and IL-1β (130). Like obesity, insufficient or inappropriate decidual recruitment and involvement of immune populations may result from endocrine disrupting chemical (EDC) such as bisphenol A (134). EDC affect estrogen and progesterone levels as well as MMPs and the activation of MMPs involves mast cell activation, which can be modified by EDC (134).

Like dNK, dMφ, and dDCs, there are uterine, placental, and fetal innate immune cells and unconventional T cells that influence development. In the 1st trimester, there are myeloid cells in many developing tissues such as microglia in the brain (135). During the 2nd trimester there are innate-like T cells in various tissues, which play a protective and homeostatic role (136). The innate-like mucosal-associated invariant T (MAIT) cells increase near term at the placenta; they may be recruited by placental chemotactic factors, and they can be anti-microbial or homeostatic (137). In the 3rd trimester with a decreased proportion of dNK, there is an increased presence of T cells (138); conventional T cells and unconventional T cell proportions, including CD4^+^ and CD8^+^ T cells fluctuate throughout pregnancy (139, 140). The innate lymphocytes include NK cells, intra-epithelial lymphocytes (IEL), lymphoid tissue-inducer (LTi), and the innate lymphoid cell subsets (ILC1, 2, and 3), which mimic the Th1, Th2, and Th17 subsets regarding some of their cytokine products, but they develop and respond faster since they bypass the need for antigen-specific stimulation (141). The ILC population is suggested to increase with implantation (142). Like dNK and dMφ subset fluctuations, as pregnancy progresses, there also is plasticity among the ILC subsets (143, 144), which seems to coincide with the local milieu of hormones, cytokine, chemokines, and growth regulatory factors. The unconventional T cells, which include invariant natural killer T (iNKT) cells, MAIT cells, and γδT cells, are enriched in barrier tissues, such as the uterus, and organs that drain these sites, such as the liver (136). In a mouse model, it was reported that intestinal microbes influence early development of thymic lymphocytes (145). Obesity affects intestinal microbiota, which can affect systemic inflammation and insulin resistance (146). Thus, obesity through changes to intestinal microbes may also affect the immune responses to the maternal-placental-fetal relationships.

The mast cell, a myeloid hematopoietic cell, has been associated with some pregnancy problems such as mast cell activation syndrome (MACS) (147). Mast cells have a diversity of functions; they can aid immune activation or immunotolerance, can produce cytokines, chemokines, neurotransmitters and neuropeptides, and can be antigen-presenting cells (APC) expressing MHC class II (58, 148, 149). Interestingly, like some macrophage subsets and other immune cells (150, 151), mast cells exist in adipose tissue and have been reported to cause chronic inflammation with obesity (152, 153). Mast cells also exist in fetuses and can influence early development of allergies with maternal IgE (154). Although mast cells can have detrimental effects on pregnancy and their activities are affected by EDC, they also play important roles such as myometrium contraction aiding birth; as for the other immune subsets affecting pregnancy, too few or too many mast cells can detrimentally affect pregnancy (134).

Since subpopulations of innate and adaptive immune cells continue to be revealed and characterized regarding immunophenotype, derivation, numbers, plasticity, and function, the safest suggestion about immune cell involvement during pregnancy with or without maternal obesity is that fluctuations in decidual, placental, and fetal immune subpopulations exist throughout the gestational period and that the proportions and numbers influence a normal vs. aberrant pregnancy. Additionally, since maternal obesity and stress from exogenous and endogenous factors can alter the balance of the immune subpopulations and affect expression of maternal-placental-fetal proteins, the mother’s condition and exposures can complicate and interfere with delivery of healthy offspring.

### Obesity, Offspring Immunity and Health

As obesity can influence inflammation and immune cell subpopulations, it also can affect offspring health for their lifetime. The potential increase in preeclampsia, gestational diabetes, hypertension, and delivery complications are all related to the oxidative stress of inflammation which imprints epigenetic changes on the developing fetus. As described earlier, metaflammation affects supply of metabolites and nutrients to the developing fetus, and it affects intrauterine programming due to maternal-placental-fetal responses to the prenatal environment, which includes increased adiposity and resulting inflammation and altered ratios of innate and adaptive immune cell subsets. Placental mRNA expressions of proinflammatory factors IL-1β, IL-8, monocyte chemoattractant protein (MCP)-1 and CXC chemokine receptor 2 (CXCR2) have been reported to be greater with maternal obesity than with non-obese women (155).

### Prematurity

Although inflammation is associated with an increase in immune cells creating an inflammatory/oxidative environment, premature oxidative stress will cause preterm birth, either *via* preterm labor or medically induced delivery to address conditions such as preeclampsia, fetal macrosomia, or poor fetal perfusion with intrauterine growth restriction, which are all increased with maternal obesity. Babies born preterm are subsequently known to be at risk for intestinal disorders, increased infection rates, respiratory disease, retinopathy, and a variety of neurodevelopmental and neurobehavioral conditions (156–158).

Sexual differences are observed in preterm infant outcomes, with morbidities in males generally poorer that in females (159). Interestingly, outside of prematurity, males display more inflammation with metabolic syndrome (160), which may be why male offspring tend to have greater prevalence of cardiovascular and neurodevelopmental disorders (161, 162). Sexually dimorphic placental responses to stresses and need for more analyses was reviewed (163). Perhaps male susceptibility to stress and inflammation partially contributes to poorer outcome in preterm males as well. The morbidities in offspring related to maternal obesity are not merely a consequence of the effects of prematurity, however. There are immunologic, metabolic, and neurologic/psychiatric sequelae that are independently associated with maternal obesity.

### Offspring Immune Function

Maternal obesity and obesogenic diets have been associated with abnormal immune function in offspring, including decreased response to infection, atopic disease, and asthma (56, 164, 165). The modifications include epigenetic and physiological programming (22, 166) that can lead to conditions such cardiovascular disease, asthma, and allergies as the neonatal immune system undergoes further exposure to environmental modulators (microbes, chemicals, and physical and psychological stressors) (21, 167, 168). In a study reviewing immunologic markers such as IgM in neonatal blood spots collected in newborn screening, this group has previously shown an association with maternal obesity and increased IgM as well as other inflammatory markers which are consistent with later immune dysregulation (169). In a mouse model, maternal high fat diet has been associated with increased incidence of Crohn’s disease-like ileitis in genetically susceptible offspring (170). Additionally, marrow adipose tissue (MAT) is endocrinologically active and contributes to bone growth and maintenance as well as hematopoiesis. Increased proportions of MAT in the marrow compartment can negatively affect hematopoiesis (171). Theoretically, the increased fetal adiposity that occurs as a result of maternal obesity may affect multiple hematopoietic cell lines, including leukocytic precursors.

### Offspring Metabolism and Obesity

**FIGURE 3:**
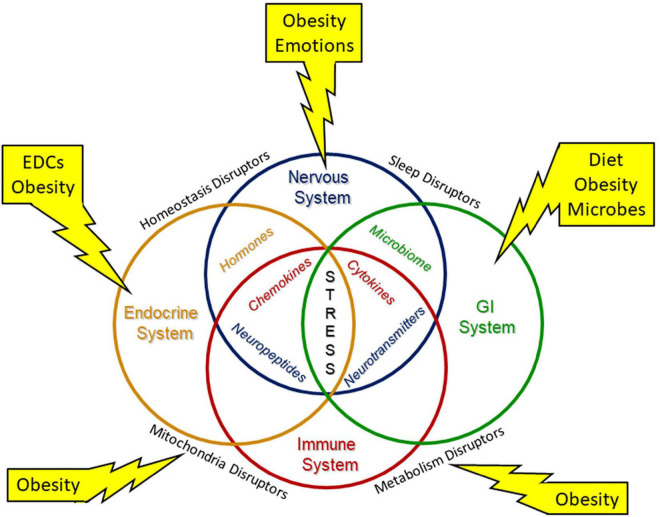
Environmental stressors modify system biology. Environmental stresses modify system biology with alterations of the neuroendocrine immune network and the gut brain axis to disrupt development. Interorgan structural and/or functional connections are needed to maintain necessary homeostasis for development and throughout life. Environmental stressors include diet, endocrine disrupting chemicals (EDCs) and other inorganic and organic chemicals, emotions, microbes, and obesity.

Maternal obesity also increases the offspring’s risk for obesity (168). Intrauterine stress has been linked over the past two decades to the development of obesity and metabolic dysfunction in offspring, both in animal and human studies (172–174). Prenatally and post-natally, obesity and other stressors can affect multiple organ systems with mutual disruption of metabolism and mitochondrial functions (Figure 3). The Maternal And Developmental Risks from Environmental and Social Stressors (MADRES) Pregnancy Cohort addressed the disproportionate increase in health issue of predominately low-income Hispanic women in urban Los Angeles (175); this report concluded that obesity and increased exposure to “obesogenic” environmental chemicals as well as higher psychosocial stress levels and less access to proper diet and health care affected both mother and offspring health. In a recent systematic review, Strain et al. (176) described multiple associations of maternal obesity and related exposures such as maternal high fat diet and maternal diabetes to metabolic consequences in offspring, including obesity, non-alcoholic fatty liver disease, and type 2 diabetes. Increasing rates of type 2 diabetes are in part attributed to intrauterine environmental exposures, such as increased inflammation and oxidative stress leading to epigenetic and other endocrine-disrupting factors (22, 151, 167). Metformin, commonly used in the treatment of type-2 diabetes, has been proposed as a treatment to lessen the obesity mediated oxidative stress effects on the placenta (119).

### Offspring Neurodevelopment

The fetal growth restriction due to detrimental placental epigenetic programming can influence inflammation including in the developing brain. Post-natally these effects can increase prevalence of cognitive impairment, autism, epilepsy, or cerebral palsy. Improved understanding of placental epigenetics and biomarkers will facilitate early predictions for likely neurodevelopment outcomes (177, 178). Some reported placental modulations such as histone modifications, DNA methylation, and hydroxymethylation, and microRNA expression might associate with metabolite levels altered with metabolic syndrome. Bangma et al. in an extensive review utilizing the ELGAN cohort and other sources, connected maternal obesity and other stressors, such as socioeconomic stress, *via* inflammatory mechanisms and placental reprogramming, to poorer neurocognitive outcomes in preterm infants (157). Maternal obesity has also been associated with increased risk for hypoxic-ischemic encephalopathy, which is significantly dictated by placental function and sufficiency (179, 180). Maternal obesity has also been associated with increased risk in offspring for multiple developmental disorders such as ADHD and autism spectrum disorder, and with psychiatric disorders such as schizophrenia and depression (181). Mechanisms include cytokine interference in neuronal development and migration (182, 183), epigenetic effects (184, 185), and fatty acid immunomodulators (174, 186, 187).

## Conclusion

In an expanding obesity pandemic, it is crucial to understand the mechanisms leading to poor outcomes for women of childbearing age, their pregnancies and the health of their offspring. We have shown that maternal obesity, an endogenous stressor, and exogenous environmental stressors contribute to abnormal oxidative and inflammatory placental changes, which then affect the fetus *via* a variety of mechanisms. Understanding these mechanisms can offer us insight into prevention, prophylaxis and treatment of this generational set of conditions. It is imperative that the medical community addresses the teratogenicity of maternal obesity and associated stresses to improve global health.

## Author Contributions

DL and MM-B was contributed to the literature review, writing, and editing of this manuscript. DL created the figures. Both authors contributed to the article and approved the submitted version.

## Conflict of Interest

The authors declare that the research was conducted in the absence of any commercial or financial relationships that could be construed as a potential conflict of interest.

## Publisher’s Note

All claims expressed in this article are solely those of the authors and do not necessarily represent those of their affiliated organizations, or those of the publisher, the editors and the reviewers. Any product that may be evaluated in this article, or claim that may be made by its manufacturer, is not guaranteed or endorsed by the publisher.

## References

[1] Driscoll AK Gregory ECW. Increases in prepregnancy obesity: United States, 2016-2019. *NCHS Data Brief.* (2020) 392:1–8. 33270551

[2] Hales CM Carroll MD Fryar CD Ogden CL. Prevalence of obesity and severe obesity among adults: United States, 2017-2018. *NCHS Data Brief.* (2020) 360:1–8. 32487284

[3] Hales CM Fryar CD Carroll MD Freedman DS Ogden CL. Trends in obesity and severe obesity prevalence in US youth and adults by sex and age, 2007-2008 to 2015-2016. *JAMA.* (2018) 319:1723–5. 10.1001/jama.2018.3060 29570750 PMC5876828

[4] Wang Y Beydoun MA Min J Xue H Kaminsky LA Cheskin LJ. Has the prevalence of overweight, obesity and central obesity levelled off in the United States? Trends, patterns, disparities, and future projections for the obesity epidemic. *Int J Epidemiol.* (2020) 49:810–23. 10.1093/ije/dyz273 32016289 PMC7394965

[5] Inoue Y Qin B Poti J Sokol R Gordon-Larsen P. Epidemiology of obesity in adults: latest trends. *Curr Obes Rep.* (2018) 7:276–88. 10.1007/s13679-018-0317-8 30155850 PMC6215729

[6] Arroyo-Johnson C Mincey KD. Obesity epidemiology worldwide. *Gastroenterol Clin North Am.* (2016) 45:571–9. 10.1016/j.gtc.2016.07.012 27837773 PMC5599163

[7] Chooi YC Ding C Magkos F. The epidemiology of obesity. *Metabolism.* (2019) 92:6–10. 30253139 10.1016/j.metabol.2018.09.005

[8] Cawley J Meyerhoefer C. The medical care costs of obesity: an instrumental variables approach. *J Health Econ.* (2012) 31:219–30. 10.1016/j.jhealeco.2011.10.003 22094013

[9] Tremmel M Gerdtham UG Nilsson PM Saha S. Economic burden of obesity: a systematic literature review. *Int J Environ Res Public Health.* (2017) 14:435. 10.3390/ijerph14040435 28422077 PMC5409636

[10] Ogden CL Carroll MD Kit BK Flegal KM. Prevalence of childhood and adult obesity in the United States, 2011-2012. *JAMA.* (2014) 311:806–14. 10.1001/jama.2014.732 24570244 PMC4770258

[11] Catalano PM Shankar K. Obesity and pregnancy: mechanisms of short term and long term adverse consequences for mother and child. *BMJ.* (2017) 356:j1. 10.1136/bmj.j1 28179267 PMC6888512

[12] Zhang C Hediger ML Albert PS Grewal J Sciscione A Grobman WA Association of maternal obesity with longitudinal ultrasonographic measures of fetal growth: findings from the NICHD fetal growth studies-singletons. *JAMA Pediatr.* (2018) 172:24–31. 10.1001/jamapediatrics.2017.3785 29131898 PMC5808867

[13] Barker DJ. The developmental origins of chronic adult disease. *Acta Paediatr.* (2004) 93:26–33. 10.1111/j.1651-2227.2004.tb00236.x 15702667

[14] Hales CM Fryar CD Carroll MD Freedman DS Aoki Y Ogden CL. Differences in obesity prevalence by demographic characteristics and urbanization level among adults in the United States, 2013-2016. *JAMA.* (2018) 319:2419–29. 10.1001/jama.2018.7270 29922829 PMC6583043

[15] Martí A Marcos A Martínez JA. Obesity and immune function relationships. *Obes Rev.* (2001) 2:131–40. 10.1046/j.1467-789x.2001.00025.x 12119664

[16] de Frel DL Atsma DE Pijl H Seidell JC Leenen PJM Dik WA The Impact of Obesity and Lifestyle on the Immune System and Susceptibility to Infections Such as COVID-19. *Front Nutr.* (2020) 7:597600. 10.3389/fnut.2020.597600 33330597 PMC7711810

[17] de Heredia FP Gómez-Martínez S Marcos A. Obesity, inflammation and the immune system. *Proc Nutr Soc.* (2012) 71:332–8. 10.1017/s0029665112000092 22429824

[18] Muscogiuri G Pugliese G Laudisio D Castellucci B Barrea L Savastano S The impact of obesity on immune response to infection: plausible mechanisms and outcomes. *Obes Rev.* (2021) 22:e13216. 10.1111/obr.13216 33719175

[19] Wolin KY Carson K Colditz GA. Obesity and cancer. *Oncologist.* (2010) 15:556–65. 20507889 10.1634/theoncologist.2009-0285 PMC3227989

[20] Godfrey KM Gluckman PD Hanson MA. Developmental origins of metabolic disease: life course and intergenerational perspectives. *Trends Endocrinol Metab.* (2010) 21:199–205. 10.1016/j.tem.2009.12.008 20080045

[21] Wilson RM Messaoudi I. The impact of maternal obesity during pregnancy on offspring immunity. *Mol Cell Endocrinol.* (2015) 418:134–42. 10.1016/j.mce.2015.07.028 26232506 PMC4674375

[22] Tomar AS Tallapragada DS Nongmaithem SS Shrestha S Yajnik CS Chandak GR. Intrauterine programming of diabetes and adiposity. *Curr Obes Rep.* (2015) 4:418–28. 10.1007/s13679-015-0175-6 26349437

[23] Svensson-Arvelund J Mehta RB Lindau R Mirrasekhian E Rodriguez-Martinez H Berg G The human fetal placenta promotes tolerance against the semiallogeneic fetus by inducing regulatory T cells and homeostatic M2 macrophages. *J Immunol.* (2015) 194:1534–44. 10.4049/jimmunol.1401536 25560409

[24] Kinder JM Stelzer IA Arck PC Way SS. Immunological implications of pregnancy-induced microchimerism. *Nat Rev Immunol.* (2017) 17:483–94. 10.1038/nri.2017.38 28480895 PMC5532073

[25] Fugazzola L Cirello V Beck-Peccoz P. Fetal microchimerism as an explanation of disease. *Nat Rev Endocrinol.* (2011) 7:89–97. 10.1038/nrendo.2010.216 21178998

[26] Stevens AM Hermes HM Rutledge JC Buyon JP Nelson JL. Myocardial-tissue-specific phenotype of maternal microchimerism in neonatal lupus congenital heart block. *Lancet.* (2003) 362:1617–23. 10.1016/S0140-6736(03)14795-2 14630442

[27] Nelson SM Sattar N Freeman DJ Walker JD Lindsay RS. Inflammation and endothelial activation is evident at birth in offspring of mothers with type 1 diabetes. *Diabetes.* (2007) 56:2697–704. 10.2337/db07-0662 17704300

[28] Liu S Diao L Huang C Li Y Zeng Y Kwak-Kim JYH. The role of decidual immune cells on human pregnancy. *J Reprod Immunol.* (2017) 124:44–53. 10.1016/j.jri.2017.10.045 29055791

[29] Ding J Zhang Y Cai X Diao L Yang C Yang J. Crosstalk between trophoblast and macrophage at the maternal-fetal interface: current status and future perspectives. *Front Immunol.* (2021) 12:758281. 10.3389/fimmu.2021.758281 34745133 PMC8566971

[30] Wang F Qualls AE Marques-Fernandez L Colucci F. Biology and pathology of the uterine microenvironment and its natural killer cells. *Cell Mol Immunol.* (2021) 18:2101–13. 10.1038/s41423-021-00739-z 34426671 PMC8429689

[31] Robertson SAPM Hunt JS. Immunology of pregnancy. In: Plant TM Zeleznik AJ editors. *Knobil and Neill’s Physiology of Reproduction.* London: Academic Press (2015). p. 1835–74.

[32] Challier JC Basu S Bintein T Minium J Hotmire K Catalano PM Obesity in pregnancy stimulates macrophage accumulation and inflammation in the placenta. *Placenta.* (2008) 29:274–81. 10.1016/j.placenta.2007.12.010 18262644 PMC4284075

[33] Frias AE Grove KL. Obesity: a transgenerational problem linked to nutrition during pregnancy. *Semin Reprod Med.* (2012) 30:472–8. 10.1055/s-0032-1328875 23074005 PMC3615704

[34] Mele J Muralimanoharan S Maloyan A Myatt L. Impaired mitochondrial function in human placenta with increased maternal adiposity. *Am J Physiol Endocrinol Metab.* (2014) 307:E419–25. 10.1152/ajpendo.00025.2014 25028397 PMC4154072

[35] Myatt L Maloyan A. Obesity and placental function. *Semin Reprod Med.* (2016) 34:42–9. 26734917 10.1055/s-0035-1570027

[36] Ospina-Serrano JS Salazar-Vargas AJ Olaya CM. Heterotopic adipose tissue in the placental parenchyma: case report. *Int J Surg Pathol.* (2021). [Online ahead of print]. 10.1177/10668969211055803 34761696

[37] Jokinen R Pirnes-Karhu S Pietiläinen KH Pirinen E. Adipose tissue NAD(+)-homeostasis, sirtuins and poly(ADP-ribose) polymerases -important players in mitochondrial metabolism and metabolic health. *Redox Biol.* (2017) 12:246–63. 10.1016/j.redox.2017.02.011 28279944 PMC5343002

[38] Ren J Wu NN Wang S Sowers JR Zhang Y. Obesity cardiomyopathy: evidence, mechanisms, and therapeutic implications. *Physiol Rev.* (2021) 101:1745–807. 10.1152/physrev.00030.2020 33949876 PMC8422427

[39] Lien YC Zhang Z Cheng Y Polyak E Sillers L Falk MJ Human placental transcriptome reveals critical alterations in inflammation and energy metabolism with fetal sex differences in spontaneous preterm birth. *Int J Mol Sci.* (2021) 22:7899. 10.3390/ijms22157899 34360662 PMC8347496

[40] Sood R Zehnder JL Druzin ML Brown PO. Gene expression patterns in human placenta. *Proc Natl Acad Sci USA.* (2006) 103:5478–83. 16567644 10.1073/pnas.0508035103 PMC1414632

[41] Cvitic S Longtine MS Hackl H Wagner K Nelson MD Desoye G The human placental sexome differs between trophoblast epithelium and villous vessel endothelium. *PLoS One.* (2013) 8:e79233. 10.1371/journal.pone.0079233 24205377 PMC3812163

[42] Coussons-Read ME. Effects of prenatal stress on pregnancy and human development: mechanisms and pathways. *Obstetr Med.* (2013) 6:52–7. 10.1177/1753495X12473751 27757157 PMC5052760

[43] Hanson MA Gluckman PD. Early developmental conditioning of later health and disease: physiology or pathophysiology?. *Physiol Rev.* (2014) 94:1027–76. 10.1152/physrev.00029.2013 25287859 PMC4187033

[44] Cattane N Räikkönen K Anniverno R Mencacci C Riva MA Pariante CM Depression, obesity and their comorbidity during pregnancy: effects on the offspring’s mental and physical health. *Mol Psychiatry.* (2021) 26:462–81. 10.1038/s41380-020-0813-6 32632208 PMC7850968

[45] Barker DJ Thornburg KL. The obstetric origins of health for a lifetime. *Clin Obstetr Gynecol.* (2013) 56:511–9. 10.1097/GRF.0b013e31829cb9ca 23787713

[46] Plana-Ripoll O Liu X Momen NC Parner E Olsen J Li J. Prenatal exposure to maternal stress following bereavement and cardiovascular disease: a nationwide population-based and sibling-matched cohort study. *Eur J Prev Cardiol.* (2016) 23:1018–28. 10.1177/2047487315585294 25952251

[47] Alexander BT Dasinger JH Intapad S. Fetal programming and cardiovascular pathology. *Compr Physiol.* (2015) 5:997–1025. 10.1002/cphy.c140036 25880521 PMC4772789

[48] Birnbaum LS Miller MF. Prenatal programming and toxicity (PPTOX) Introduction. *Endocrinology.* (2015) 156:3405–7. 10.1210/en.2015-1458 26241073 PMC4588826

[49] Linnet KM Dalsgaard S Obel C Wisborg K Henriksen TB Rodriguez A Maternal lifestyle factors in pregnancy risk of attention deficit hyperactivity disorder and associated behaviors: review of the current evidence. *Am J Psychiatry.* (2003) 160:1028–40. 10.1176/appi.ajp.160.6.1028 12777257

[50] Beversdorf DQ Manning SE Hillier A Anderson SL Nordgren RE Walters SE Timing of prenatal stressors and autism. *J Autism Dev Disord.* (2005) 35:471–8. 10.1007/s10803-005-5037-8 16134032

[51] Talge NM Neal C Glover V. Antenatal maternal stress and long-term effects on child neurodevelopment: how and why?. *J Child Psychol Psychiatry.* (2007) 48:245–61. 10.1111/j.1469-7610.2006.01714.x 17355398 PMC11016282

[52] Kinney DK Munir KM Crowley DJ Miller AM. Prenatal stress and risk for autism. *Neurosci Biobehav Rev.* (2008) 32:1519–32. 10.1016/j.neubiorev.2008.06.004 18598714 PMC2632594

[53] Howell KR Powell TL. Effects of maternal obesity on placental function and fetal development. *Reproduction.* (2017) 153:R97–108. 10.1530/REP-16-0495 27864335 PMC5432127

[54] Gregor MF Hotamisligil GS. Inflammatory mechanisms in obesity. *Annu Rev Immunol.* (2011) 29:415–45. 21219177 10.1146/annurev-immunol-031210-101322

[55] Pantham P Aye IL Powell TL. Inflammation in maternal obesity and gestational diabetes mellitus. *Placenta.* (2015) 36:709–15. 10.1016/j.placenta.2015.04.006 25972077 PMC4466145

[56] Myles IA Fontecilla NM Janelsins BM Vithayathil PJ Segre JA Datta SK. Parental dietary fat intake alters offspring microbiome and immunity. *J Immunol.* (2013) 191:3200–9. 10.4049/jimmunol.1301057 23935191 PMC3831371

[57] Farley D Tejero ME Comuzzie AG Higgins PB Cox L Werner SL Feto-placental adaptations to maternal obesity in the baboon. *Placenta.* (2009) 30:752–60. 10.1016/j.placenta.2009.06.007 19632719 PMC3011231

[58] Caslin HL Bhanot M Bolus WR Hasty AH. Adipose tissue macrophages: unique polarization and bioenergetics in obesity. *Immunol Rev.* (2020) 295:101–13. 10.1111/imr.12853 32237081 PMC8015437

[59] Schwenke M Knöfler M Velicky P Weimar CH Kruse M Samalecos A Control of human endometrial stromal cell motility by PDGF-BB, HB-EGF and trophoblast-secreted factors. *PLoS One.* (2013) 8:e54336. 10.1371/journal.pone.0054336 23349855 PMC3549986

[60] Du MR Wang SC Li DJ. The integrative roles of chemokines at the maternal-fetal interface in early pregnancy. *Cell Mol Immunol.* (2014) 11:438–48. 10.1038/cmi.2014.68 25109684 PMC4197212

[61] Aye IL Lager S Ramirez VI Gaccioli F Dudley DJ Jansson T Increasing maternal body mass index is associated with systemic inflammation in the mother and the activation of distinct placental inflammatory pathways. *Biol Reprod.* (2014) 90:129. 10.1095/biolreprod.113.116186 24759787 PMC4094003

[62] Aye IL Rosario FJ Powell TL Jansson T. Adiponectin supplementation in pregnant mice prevents the adverse effects of maternal obesity on placental function and fetal growth. *Proc Natl Acad Sci USA.* (2015) 112:12858–63. 10.1073/pnas.1515484112 26417088 PMC4611638

[63] Brunst KJ Sanchez Guerra M Gennings C Hacker M Jara C Bosquet Enlow M Maternal lifetime stress and prenatal psychological functioning and decreased placental mitochondrial DNA copy number in the PRISM study. *Am J Epidemiol.* (2017) 186:1227–36. 10.1093/aje/kwx183 28595325 PMC5859981

[64] Schoots MH Gordijn SJ Scherjon SA van Goor H Hillebrands JL. Oxidative stress in placental pathology. *Placenta.* (2018) 69:153–61. 10.1016/j.placenta.2018.03.003 29622278

[65] Burton GJ Jauniaux E. Pathophysiology of placental-derived fetal growth restriction. *Am J Obstetr Gynecol.* (2018) 218:S745–61. 10.1016/j.ajog.2017.11.577 29422210

[66] Abelius MS Janefjord C Ernerudh J Berg G Matthiesen L Duchén K The placental immune milieu is characterized by a Th2- and anti-inflammatory transcription profile, regardless of maternal allergy, and associates with neonatal immunity. *Am J Reprod Immunol.* (2015) 73:445–59. 10.1111/aji.12350 25491384

[67] Aufdenblatten M Baumann M Raio L Dick B Frey BM Schneider H Prematurity is related to high placental cortisol in preeclampsia. *Pediatr Res.* (2009) 65:198–202. 10.1203/PDR.0b013e31818d6c24 19047954

[68] Thornburg KL Boone-Heinonen J Valent AM. Social determinants of placental health and future disease risks for babies. *Obstetr Gynecol Clin North Am.* (2020) 47:1–15. 10.1016/j.ogc.2019.11.002 32008662 PMC7732260

[69] Rana S Lemoine E Granger JP Karumanchi SA. Preeclampsia: pathophysiology, Challenges, and Perspectives. *Circ Res.* (2019) 124:1094–112. 10.1161/circresaha.118.313276 30920918

[70] Staff AC. The two-stage placental model of preeclampsia: an update. *J Reprod Immunol.* (2019) 134-135:1–10. 10.1016/j.jri.2019.07.004 31301487

[71] Austdal M Thomsen LC Tangerås LH Skei B Mathew S Bjørge L Metabolic profiles of placenta in preeclampsia using HR-MAS MRS metabolomics. *Placenta.* (2015) 36:1455–62. 10.1016/j.placenta.2015.10.019 26582504

[72] Wheeler KC Jena MK Pradhan BS Nayak N Das S Hsu CD VEGF may contribute to macrophage recruitment and M2 polarization in the decidua. *PLoS One.* (2018) 13:e0191040. 10.1371/journal.pone.0191040 29324807 PMC5764356

[73] Ives CW Sinkey R Rajapreyar I Tita ATN Oparil S. Preeclampsia-pathophysiology and clinical presentations: JACC state-of-the-art review. *J Am Coll Cardiol.* (2020) 76:1690–702. 10.1016/j.jacc.2020.08.014 33004135

[74] Benton SJ Ly C Vukovic S Bainbridge SA. Andrée Gruslin award lecture: metabolomics as an important modality to better understand preeclampsia. *Placenta.* (2017) 60:S32–40. 10.1016/j.placenta.2016.11.006 27889063

[75] Shekhawat PS Matern D Strauss AW. Fetal fatty acid oxidation disorders, their effect on maternal health and neonatal outcome: impact of expanded newborn screening on their diagnosis and management. *Pediatr Res.* (2005) 57:78r–86r. 10.1203/01.PDR.0000159631.63843.3E 15817498 PMC3582391

[76] Houten SM Wanders RJA Ranea-Robles P. Metabolic interactions between peroxisomes and mitochondria with a special focus on acylcarnitine metabolism. *Biochim Biophys Acta Mol Basis Dis.* (2020) 1866:165720. 10.1016/j.bbadis.2020.165720 32057943 PMC7146961

[77] Fisher JJ Bartho LA Perkins AV Holland OJ. Placental mitochondria and reactive oxygen species in the physiology and pathophysiology of pregnancy. *Clin Exp Pharmacol Physiol.* (2020) 47:176–84. 10.1111/1440-1681.13172 31469913

[78] Al-Gubory KH Fowler PA Garrel C. The roles of cellular reactive oxygen species, oxidative stress and antioxidants in pregnancy outcomes. *Int J Biochem Cell Biol.* (2010) 42:1634–50. 10.1016/j.biocel.2010.06.001 20601089

[79] Marín R Chiarello DI Abad C Rojas D Toledo F Sobrevia L. Oxidative stress and mitochondrial dysfunction in early-onset and late-onset preeclampsia. *Biochim Biophys Acta Mol Basis Dis.* (2020) 1866:165961. 10.1016/j.bbadis.2020.165961 32916282

[80] Gusar V Ganichkina M Chagovets V Kan N Sukhikh G. MiRNAs regulating oxidative stress: a correlation with doppler sonography of uteroplacental complex and clinical state assessments of newborns in fetal growth restriction. *J Clin Med.* (2020) 9:3227. 10.3390/jcm9103227 33050114 PMC7650709

[81] Zhang Y Zhao W Xu H Hu M Guo X Jia W Hyperandrogenism and insulin resistance-induced fetal loss: evidence for placental mitochondrial abnormalities and elevated reactive oxygen species production in pregnant rats that mimic the clinical features of polycystic ovary syndrome. *J Physiol.* (2019) 597:3927–50. 10.1113/JP277879 31206177

[82] Duthie L Reynolds RM. Changes in the maternal hypothalamic-pituitary-adrenal axis in pregnancy and postpartum: influences on maternal and fetal outcomes. *Neuroendocrinology.* (2013) 98:106–15. 10.1159/000354702 23969897

[83] Stirrat LI O’Reilly JR Barr SM Andrew R Riley SC Howie AF Decreased maternal hypothalamic-pituitary-adrenal axis activity in very severely obese pregnancy: associations with birthweight and gestation at delivery. *Psychoneuroendocrinology.* (2016) 63:135–43. 10.1016/j.psyneuen.2015.09.019 26444587

[84] Johns EC Denison FC Reynolds RM. The impact of maternal obesity in pregnancy on placental glucocorticoid and macronutrient transport and metabolism. *Biochim Biophys Acta Mol Basis Dis.* (2020) 1866:165374. 10.1016/j.bbadis.2018.12.025 30684643

[85] Hirst JJ Cumberland AL Shaw JC Bennett GA Kelleher MA Walker DW Loss of neurosteroid-mediated protection following stress during fetal life. *J Steroid Biochem Mol Biol.* (2016) 160:181–8. 10.1016/j.jsbmb.2015.09.012 26365557

[86] Braun T Challis JR Newnham JP Sloboda DM. Early-life glucocorticoid exposure: the hypothalamic-pituitary-adrenal axis, placental function, and long-term disease risk. *Endocr Rev.* (2013) 34:885–916. 10.1210/er.2013-1012 23970762

[87] Fowden AL Forhead AJ. Glucocorticoids as regulatory signals during intrauterine development. *Exp Physiol.* (2015) 100:1477–87. 10.1113/EP085212 26040783

[88] Paquette AG Lester BM Lesseur C Armstrong DA Guerin DJ Appleton AA Placental epigenetic patterning of glucocorticoid response genes is associated with infant neurodevelopment. *Epigenomics.* (2015) 7:767–79. 10.2217/epi.15.28 26343289 PMC4772971

[89] Harris A Seckl J. Glucocorticoids, prenatal stress and the programming of disease. *Horm Behav.* (2011) 59:279–89. 10.1016/j.yhbeh.2010.06.007 20591431

[90] Seckl JR Holmes MC. Mechanisms of disease: glucocorticoids, their placental metabolism and fetal ‘programming’ of adult pathophysiology. *Nat Clin Pract Endocrinol Metab.* (2007) 3:479–88. 10.1038/ncpendmet0515 17515892

[91] Reynolds RM. Programming effects of glucocorticoids. *Clin Obstetr Gynecol.* (2013) 56:602–9. 10.1097/grf.0b013e31829939f7 23722920

[92] McTernan CL Draper N Nicholson H Chalder SM Driver P Hewison M Reduced placental 11beta-hydroxysteroid dehydrogenase type 2 mRNA levels in human pregnancies complicated by intrauterine growth restriction: an analysis of possible mechanisms. *J Clin Endocrinol Metab.* (2001) 86:4979–83. 10.1210/jcem.86.10.7893 11600574

[93] Seth S Lewis AJ Saffery R Lappas M Galbally M. Maternal prenatal mental health and placental 11β-HSD2 gene expression: initial findings from the mercy pregnancy and emotional wellbeing study. *Int J Mol Sci.* (2015) 16:27482–96. 10.3390/ijms161126034 26593902 PMC4661892

[94] Bronson SL Bale TL. The placenta as a mediator of stress effects on neurodevelopmental reprogramming. *Neuropsychopharmacology.* (2016) 41:207–18. 10.1038/npp.2015.231 26250599 PMC4677129

[95] Solano ME Arck PC. Steroids, pregnancy and fetal development. *Front Immunol.* (2019) 10:3017. 10.3389/fimmu.2019.03017 32038609 PMC6987319

[96] Rakers F Rupprecht S Dreiling M Bergmeier C Witte OW Schwab M. Transfer of maternal psychosocial stress to the fetus. *Neurosci Biobehav Rev.* (2017). [Online ahead of print]. 10.1016/j.neubiorev.2017.02.019 28237726

[97] Robinson DP Klein SL. Pregnancy and pregnancy-associated hormones alter immune responses and disease pathogenesis. *Horm Behav.* (2012) 62:263–71. 10.1016/j.yhbeh.2012.02.023 22406114 PMC3376705

[98] Kumar P Magon N. Hormones in pregnancy. *Niger Med J.* (2012) 53:179–83. 23661874 10.4103/0300-1652.107549 PMC3640235

[99] Jarmund AH Giskeødegård GF Ryssdal M Steinkjer B Stokkeland LMT Madssen TS Cytokine patterns in maternal serum from first trimester to term and beyond. *Front Immunol.* (2021) 12:752660. 10.3389/fimmu.2021.752660 34721426 PMC8552528

[100] Faas MM de Vos P. Uterine NK cells and macrophages in pregnancy. *Placenta.* (2017) 56:44–52. 10.1016/j.placenta.2017.03.001 28284455

[101] Schumacher A Sharkey DJ Robertson SA Zenclussen AC. Immune cells at the fetomaternal interface: how the microenvironment modulates immune cells to foster fetal development. *J Immunol.* (2018) 201:325–34. 10.4049/jimmunol.1800058 29987001

[102] Zhang YH He M Wang Y Liao AH. Modulators of the balance between M1 and M2 macrophages during pregnancy. *Front Immunol.* (2017) 8:120. 10.3389/fimmu.2017.00120 28232836 PMC5299000

[103] Yao Y Xu XH Jin L. Macrophage polarization in physiological and pathological pregnancy. *Front Immunol.* (2019) 10:792. 10.3389/fimmu.2019.00792 31037072 PMC6476302

[104] Kämmerer U Eggert AO Kapp M McLellan AD Geijtenbeek TB Dietl J Unique appearance of proliferating antigen-presenting cells expressing DC-SIGN (CD209) in the decidua of early human pregnancy. *Am J Pathol.* (2003) 162:887–96. 10.1016/S0002-9440(10)63884-9 12598322 PMC1868095

[105] Renaud SJ Graham CH. The role of macrophages in utero-placental interactions during normal and pathological pregnancy. *Immunol Investig.* (2008) 37:535–64. 10.1080/08820130802191375 18716937

[106] Hsu P Santner-Nanan B Dahlstrom JE Fadia M Chandra A Peek M Altered decidual DC-SIGN+ antigen-presenting cells and impaired regulatory T-cell induction in preeclampsia. *Am J Pathol.* (2012) 181:2149–60. 10.1016/j.ajpath.2012.08.032 23063509

[107] Murata Y Shimamura T Hamuro J. The polarization of T(h)1/T(h)2 balance is dependent on the intracellular thiol redox status of macrophages due to the distinctive cytokine production. *Int Immunol.* (2002) 14:201–12. 10.1093/intimm/14.2.201 11809739

[108] Lynes MA Hidalgo J Manso Y Devisscher L Laukens D Lawrence DA. Metallothionein and stress combine to affect multiple organ systems. *Cell Stress Chaperones.* (2014) 19:605–11. 10.1007/s12192-014-0501-z 24584987 PMC4147071

[109] Alcala M Gutierrez-Vega S Castro E Guzman-Gutiérrez E Ramos-Álvarez MP Viana M. Antioxidants and oxidative stress: focus in obese pregnancies. *Front Physiol.* (2018) 9:1569. 10.3389/fphys.2018.01569 30459642 PMC6232303

[110] Alcalá M Sánchez-Vera I Sevillano J Herrero L Serra D Ramos MP Vitamin E reduces adipose tissue fibrosis, inflammation, and oxidative stress and improves metabolic profile in obesity. *Obesity.* (2015) 23:1598–606. 10.1002/oby.21135 26148343

[111] Vranić L Mikolašević I Milić S. Vitamin D deficiency: consequence or cause of obesity? *Medicina.* (2019) 55:541. 10.3390/medicina55090541 31466220 PMC6780345

[112] Thomas-Valdés S Tostes M Anunciação PC da Silva BP Sant’Ana HMP. Association between vitamin deficiency and metabolic disorders related to obesity. *Crit Rev Food Sci Nutr.* (2017) 57:3332–43. 10.1080/10408398.2015.1117413 26745150

[113] Garcia-Diaz DF Lopez-Legarrea P Quintero P Martinez JA. Vitamin C in the treatment and/or prevention of obesity. *J Nutr Sci Vitaminol.* (2014) 60:367–79. 10.3177/jnsv.60.367 25866299

[114] Sen S Iyer C Meydani SN. Obesity during pregnancy alters maternal oxidant balance and micronutrient status. *J Perinatol.* (2014) 34:105–11. 10.1038/jp.2013.153 24355940

[115] Ivanov D Mazzoccoli G Anderson G Linkova N Dyatlova A Mironova E Melatonin, its beneficial effects on embryogenesis from mitigating oxidative stress to regulating gene expression. *Int J Mol Sci.* (2021) 22:5885. 10.3390/ijms22115885 34070944 PMC8198864

[116] Ivanov DO Evsyukova II Mazzoccoli G Anderson G Polyakova VO Kvetnoy IM The role of prenatal melatonin in the regulation of childhood obesity. *Biology.* (2020) 9:42. 10.3390/biology9040072 32260529 PMC7235795

[117] Ivanov DO Evsyukova II Mironova ES Polyakova VO Kvetnoy IM Nasyrov RA. Maternal melatonin deficiency leads to endocrine pathologies in children in early ontogenesis. *Int J Mol Sci.* (2021) 22:2058. 10.3390/ijms22042058 33669686 PMC7922827

[118] Han L Wang H Li L Li X Ge J Reiter RJ Melatonin protects against maternal obesity-associated oxidative stress and meiotic defects in oocytes via the SIRT3-SOD2-dependent pathway. *J Pineal Res.* (2017) 63:e12431. 10.1111/jpi.12431 28658527

[119] Bedell S Hutson J de Vrijer B Eastabrook G. Effects of maternal obesity and gestational diabetes mellitus on the placenta: current knowledge and targets for therapeutic interventions. *Curr Vasc Pharmacol.* (2021) 19:176–92. 10.2174/1570161118666200616144512 32543363

[120] Nagamatsu T Schust DJ. The contribution of macrophages to normal and pathological pregnancies. *Am J Reprod Immunol.* (2010) 63:460–71. 10.1111/j.1600-0897.2010.00813.x 20163399

[121] Brown MB von Chamier M Allam AB Reyes L. M1/M2 macrophage polarity in normal and complicated pregnancy. *Front Immunol.* (2014) 5:606. 10.3389/fimmu.2014.00606 25505471 PMC4241843

[122] Medeiros LT Peraçoli JC Bannwart-Castro CF Romão M Weel IC Golim MA Monocytes from pregnant women with pre-eclampsia are polarized to a M1 phenotype. *Am J Reprod Immunol.* (2014):72:5–13. 10.1111/aji.12222 24689463

[123] Melgert BN Spaans F Borghuis T Klok PA Groen B Bolt A Pregnancy and preeclampsia affect monocyte subsets in humans and rats. *PLoS One.* (2012) 7:e45229. 10.1371/journal.pone.0045229 23028864 PMC3441708

[124] Schonkeren D van der Hoorn ML Khedoe P Swings G van Beelen E Claas F Differential distribution and phenotype of decidual macrophages in preeclamptic versus control pregnancies. *Am J Pathol.* (2011) 178:709–17. 10.1016/j.ajpath.2010.10.011 21281803 PMC3069820

[125] Gomez-Lopez N StLouis D Lehr MA Sanchez-Rodriguez EN Arenas-Hernandez M. Immune cells in term and preterm labor. *Cell Mol Immunol.* (2014) 11:571–81. 10.1038/cmi.2014.46 24954221 PMC4220837

[126] Huhn O Zhao X Esposito L Moffett A Colucci F Sharkey AM. How do uterine natural killer and innate lymphoid cells contribute to successful pregnancy?. *Front Immunol.* (2021) 12:607669. 10.3389/fimmu.2021.607669 34234770 PMC8256162

[127] Zhou Y Fu B Xu X Zhang J Tong X Wang Y PBX1 expression in uterine natural killer cells drives fetal growth. *Sci Transl Med.* (2020) 12:eaax1798. 10.1126/scitranslmed.aax1798 32238574

[128] Fu B Zhou Y Ni X Tong X Xu X Dong Z Natural killer cells promote fetal development through the secretion of growth-promoting factors. *Immunity.* (2017) 47:1100–13.e6. 10.1016/j.immuni.2017.11.018 29262349

[129] Jiang X Du MR Li M Wang H. Three macrophage subsets are identified in the uterus during early human pregnancy. *Cell Mol Immunol.* (2018) 15:1027–37. 10.1038/s41423-018-0008-0 29618777 PMC6269440

[130] Sun F Wang S Du M. Functional regulation of decidual macrophages during pregnancy. *J Reprod Immunol.* (2021) 143:103264. 10.1016/j.jri.2020.103264 33360717

[131] Balan S Saxena M Bhardwaj N. Dendritic cell subsets and locations. *Int Rev Cell Mol Biol.* (2019) 348:1–68. 10.1016/bs.ircmb.2019.07.004 31810551

[132] van Wigcheren GF Roelofs D Figdor CG Flórez-Grau G. Three distinct tolerogenic CD14(+) myeloid cell types to actively manage autoimmune disease: opportunities and challenges. *J Autoimmun.* (2021) 120:102645. 10.1016/j.jaut.2021.102645 33901801

[133] Wei R Lai N Zhao L Zhang Z Zhu X Guo Q Dendritic cells in pregnancy and pregnancy-associated diseases. *Biomed Pharmacother.* (2021) 133:110921. 10.1016/j.biopha.2020.110921 33378991

[134] Meyer N Zenclussen AC. Immune cells in the uterine remodeling: are they the target of endocrine disrupting chemicals?. *Front Immunol.* (2020) 11:246. 10.3389/fimmu.2020.00246 32140155 PMC7043066

[135] Verney C Monier A Fallet-Bianco C Gressens P. Early microglial colonization of the human forebrain and possible involvement in periventricular white-matter injury of preterm infants. *J Anat.* (2010) 217:436–48. 10.1111/j.1469-7580.2010.01245.x 20557401 PMC2992419

[136] Constantinides MG Belkaid Y. Early-life imprinting of unconventional T cells and tissue homeostasis. *Science.* (2021) 374:eabf0095. 10.1126/science.abf0095 34882451 PMC8697520

[137] Kaipe H Raffetseder J Ernerudh J Solders M Tiblad EMAIT. Cells at the Fetal-Maternal Interface During Pregnancy. *Front Immunol.* (2020) 11:1788. 10.3389/fimmu.2020.01788 32973750 PMC7466580

[138] Williams PJ Searle RF Robson SC Innes BA Bulmer JN. Decidual leucocyte populations in early to late gestation normal human pregnancy. *J Reprod Immunol.* (2009) 82:24–31. 10.1016/j.jri.2009.08.001 19732959

[139] Chapman JC Chapman FM Michael SD. The production of alpha/beta and gamma/delta double negative (DN) T-cells and their role in the maintenance of pregnancy. *Reprod Biol Endocrinol.* (2015) 13:73. 10.1186/s12958-015-0073-5 26164866 PMC4499209

[140] Hardardottir L Bazzano MV Glau L Gattinoni L Köninger A Tolosa E The new old CD8+ T cells in the immune paradox of pregnancy. *Front Immunol.* (2021) 12:765730. 10.3389/fimmu.2021.765730 34868016 PMC8635142

[141] Simoni Y Newell EW. Dissecting human ILC heterogeneity: more than just three subsets. *Immunology.* (2018) 153:297–303. 10.1111/imm.12862 29140572 PMC5795188

[142] Monin L Whettlock EM Male V. Immune responses in the human female reproductive tract. *Immunology.* (2020) 160:106–15. 10.1111/imm.13136 31630394 PMC7218661

[143] Bal SM Golebski K Spits H. Plasticity of innate lymphoid cell subsets. *Nat Rev Immunol.* (2020) 20:552–65. 10.1038/s41577-020-0282-9 32107466

[144] Vacca P Chiossone L Mingari MC Moretta L. Heterogeneity of NK cells and other innate lymphoid cells in human and murine decidua. *Front Immunol.* (2019) 10:170. 10.3389/fimmu.2019.00170 30800126 PMC6375891

[145] Ennamorati M Vasudevan C Clerkin K Halvorsen S Verma S Ibrahim S Intestinal microbes influence development of thymic lymphocytes in early life. *Proc Natl. Acad Sci USA.* (2020) 117:2570–8. 10.1073/pnas.1915047117 31964813 PMC7007548

[146] Gomes AC Hoffmann C Mota JF. The human gut microbiota: metabolism and perspective in obesity. *Gut Microbes.* (2018) 9:308–25. 10.1080/19490976.2018.1465157 29667480 PMC6219651

[147] Dorff SR Afrin LB. Mast cell activation syndrome in pregnancy, delivery, postpartum and lactation: a narrative review. *J Obstetr Gynaecol.* (2020) 40:889–901. 10.1080/01443615.2019.1674259 32148151

[148] da Silva EZ Jamur MC Oliver C. Mast cell function: a new vision of an old cell. *J Histochem Cytochem.* (2014) 62:698–738. 10.1369/0022155414545334 25062998 PMC4230976

[149] Theoharides TC Tsilioni I Ren H. Recent advances in our understanding of mast cell activation - or should it be mast cell mediator disorders?. *Expert Rev Clin Immunol.* (2019) 15:639–56. 10.1080/1744666X.2019.1596800 30884251 PMC7003574

[150] Zatterale F Longo M Naderi J Raciti GA Desiderio A Miele C Chronic adipose tissue inflammation linking obesity to insulin resistance and type 2 diabetes. *Front Physiol.* (2019) 10:1607. 10.3389/fphys.2019.01607 32063863 PMC7000657

[151] Lee BC Lee J. Cellular and molecular players in adipose tissue inflammation in the development of obesity-induced insulin resistance. *Biochim Biophys Acta.* (2014) 1842:446–62. 10.1016/j.bbadis.2013.05.017 23707515 PMC3800253

[152] Żelechowska P Agier J Kozłowska E Brzeziñska-Błaszczyk E. Mast cells participate in chronic low-grade inflammation within adipose tissue. *Obes Rev.* (2018) 19:686–97. 10.1111/obr.12670 29334696

[153] Gurung P Moussa K Adams-Huet B Devaraj S Jialal I. Increased mast cell abundance in adipose tissue of metabolic syndrome: relevance to the proinflammatory state and increased adipose tissue fibrosis. *Am J Physiol Endocrinol Metab.* (2019) 316:E504–9. 10.1152/ajpendo.00462.2018 30620639

[154] Msallam R Balla J Rathore APS Kared H Malleret B Saron WAA Fetal mast cells mediate postnatal allergic responses dependent on maternal IgE. *Science.* (2020) 370:941–50. 10.1126/science.aba0864 33122426

[155] Roberts KA Riley SC Reynolds RM Barr S Evans M Statham A Placental structure and inflammation in pregnancies associated with obesity. *Placenta.* (2011) 32:247–54. 10.1016/j.placenta.2010.12.023 21232790

[156] Oldenburg KS O’Shea TM Fry RC. Genetic and epigenetic factors and early life inflammation as predictors of neurodevelopmental outcomes. *Semin Fetal Neonatal Med.* (2020) 25:101115. 10.1016/j.siny.2020.101115 32444251 PMC7363586

[157] Bangma JT Hartwell H Santos HP Jr. O’Shea TM Fry RC. Placental programming, perinatal inflammation, and neurodevelopment impairment among those born extremely preterm. *Pediatr Res.* (2021) 89:326–35. 10.1038/s41390-020-01236-1 33184498 PMC7658618

[158] Platt MJ. Outcomes in preterm infants. *Public Health.* (2014) 128:399–403. 24794180 10.1016/j.puhe.2014.03.010

[159] Garfinkle J Yoon EW Alvaro R Nwaesei C Claveau M Lee SK Trends in sex-specific differences in outcomes in extreme preterms: progress or natural barriers?. *Arch Dis Child Fetal Neonatal Ed.* (2020) 105:158–63. 10.1136/archdischild-2018-316399 31186268

[160] Ter Horst R van den Munckhof ICL Schraa K Aguirre-Gamboa R Jaeger M Smeekens SP Sex-specific regulation of inflammation and metabolic syndrome in obesity. *Arterioscler Thromb vasc Biol.* (2020) 40:1787–800. 10.1161/ATVBAHA.120.314508 32460579 PMC7310302

[161] Thornburg KL O’Tierney PF Louey S. Review: the placenta is a programming agent for cardiovascular disease. *Placenta.* (2010) 31:S54–9. 10.1016/j.placenta.2010.01.002 20149453 PMC2846089

[162] Hsiao EY Patterson PH. Placental regulation of maternal-fetal interactions and brain development. *Dev Neurobiol.* (2012) 72:1317–26. 10.1002/dneu.22045 22753006

[163] Rosenfeld CS. Sex-specific placental responses in fetal development. *Endocrinology.* (2015) 156:3422–34. 10.1210/en.2015-1227 26241064 PMC4588817

[164] Forno E Young OM Kumar R Simhan H Celedón JC. Maternal obesity in pregnancy, gestational weight gain, and risk of childhood asthma. *Pediatrics.* (2014) 134:e535–46. 10.1542/peds.2014-0439 25049351 PMC4187236

[165] Harpsøe MC Basit S Bager P Wohlfahrt J Benn CS Nøhr EA Maternal obesity, gestational weight gain, and risk of asthma and atopic disease in offspring: a study within the Danish National Birth Cohort. *J Allergy Clin Immunol.* (2013) 131:1033–40. 10.1016/j.jaci.2012.09.008 23122630

[166] Yajnik CS. Transmission of obesity-adiposity and related disorders from the mother to the baby. *Ann Nutr Metab.* (2014) 64:8–17. 10.1159/000362608 25059801

[167] Rughani A Friedman JE Tryggestad JB. Type 2 diabetes in youth: the role of early life exposures. *Curr Diabetes Rep.* (2020) 20:45. 10.1007/s11892-020-01328-6 32767148

[168] Godfrey KM Reynolds RM Prescott SL Nyirenda M Jaddoe VW Eriksson JG Influence of maternal obesity on the long-term health of offspring. *Lancet Diabetes Endocrinol.* (2017) 5:53–64. 10.1016/S2213-8587(16)30107-3 27743978 PMC5245733

[169] Broadney MM Chahal N Michels KA McLain AC Ghassabian A Lawrence DA Impact of parental obesity on neonatal markers of inflammation and immune response. *Int J Obes.* (2017) 41:30–7. 10.1038/ijo.2016.187 27780976 PMC5209273

[170] Gruber L Hemmerling J Schüppel V Müller M Boekschoten MV Haller D. maternal high-fat diet accelerates development of crohn’s disease-like Ileitis in TNFΔARE/WT offspring. *Inflamm Bowel Dis.* (2015) 21:2016–25. 10.1097/MIB.0000000000000465 26284294

[171] Slayton WB Juul SE Calhoun DA Li Y Braylan RC Christensen RD. Hematopoiesis in the liver and marrow of human fetuses at 5 to 16 weeks postconception: quantitative assessment of macrophage and neutrophil populations. *Pediatr Res.* (1998) 43:774–82. 10.1203/00006450-199806000-00010 9621987

[172] Dalrymple KV Thompson JMD Begum S Godfrey KM Poston L Seed PT Relationships of maternal body mass index and plasma biomarkers with childhood body mass index and adiposity at 6 years: the children of SCOPE study. *Pediatr Obes.* (2019) 14:e12537. 10.1111/ijpo.12537 31232532 PMC6731120

[173] Entringer S Wadhwa PD. Developmental programming of obesity and metabolic dysfunction: role of prenatal stress and stress biology. *Nestle Nutr Inst Workshop Ser.* (2013) 74:107–20. 10.1159/000348454 23887109 PMC4159714

[174] Jarvie E Hauguel-de-Mouzon S Nelson SM Sattar N Catalano PM Freeman DJ. Lipotoxicity in obese pregnancy and its potential role in adverse pregnancy outcome and obesity in the offspring. *Clin Sci.* (2010) 119:123–9. 10.1042/CS20090640 20443782 PMC2860697

[175] Bastain TM Chavez T Habre R Girguis MS Grubbs B Toledo-Corral C Study design, protocol and profile of the maternal and developmental risks from environmental and social stressors (MADRES) pregnancy cohort: a prospective cohort study in predominantly low-income hispanic women in urban los angeles. *BMC Pregnancy Childbirth.* (2019) 19:189. 10.1186/s12884-019-2330-7 31146718 PMC6543670

[176] Strain J Spaans F Serhan M Davidge ST Connor KL. Programming of weight and obesity across the lifecourse by the maternal metabolic exposome: a systematic review. *Mol Aspects Med.* (2021). [Online ahead of print]. 10.1016/j.mam.2021.100986 34167845

[177] Hodyl NA Aboustate N Bianco-Miotto T Roberts CT Clifton VL Stark MJ. Child neurodevelopmental outcomes following preterm and term birth: what can the placenta tell us?. *Placenta.* (2017) 57:79–86. 10.1016/j.placenta.2017.06.009 28864022

[178] Reichetzeder C. Overweight and obesity in pregnancy: their impact on epigenetics. *Eur J Clin Nutr.* (2021) 75:1710–22. 10.1038/s41430-021-00905-6 34230629 PMC8636269

[179] Barrois M Patkai J Delorme P Chollat C Goffinet F Le Ray C. Factors associated with neonatal hypoxic ischemic encephalopathy in infants with an umbilical artery pH less than 7.00. *Eur J Obstetr Gynecol Reprod Biol.* (2019) 236:69–74. 10.1016/j.ejogrb.2019.02.009 30884338

[180] Khalak R Horgan M. Association of maternal obesity and neonatal hypoxic ischemic encephalopathy. *J Perinatol.* (2020) 40:174–5. 10.1038/s41372-019-0559-7 31748656

[181] Rivera HM Christiansen KJ Sullivan EL. The role of maternal obesity in the risk of neuropsychiatric disorders. *Front Neurosci.* (2015) 9:194. 10.3389/fnins.2015.00194 26150767 PMC4471351

[182] Sanders TR Kim DW Glendining KA Jasoni CL. Maternal obesity and IL-6 lead to aberrant developmental gene expression and deregulated neurite growth in the fetal arcuate nucleus. *Endocrinology.* (2014) 155:2566–77. 10.1210/en.2013-1968 24773340

[183] Nolan AM Collins LM Wyatt SL Gutierrez H O’Keeffe GW. The neurite growth inhibitory effects of soluble TNFα on developing sympathetic neurons are dependent on developmental age. *Differentiation.* (2014) 88:124–30. 10.1016/j.diff.2014.12.006 25582843

[184] Mitsuya K Parker AN Liu L Ruan J Vissers MCM Myatt L. Alterations in the placental methylome with maternal obesity and evidence for metabolic regulation. *PLoS One.* (2017) 12:e0186115. 10.1371/journal.pone.0186115 29045485 PMC5646778

[185] Ramaiyan B Talahalli RR. Dietary unsaturated fatty acids modulate maternal dyslipidemia-induced DNA methylation and histone acetylation in placenta and fetal liver in rats. *Lipids.* (2018) 53:581–8. 10.1002/lipd.12074 30203512

[186] Zhu MJ Ma Y Long NM Du M Ford SP. Maternal obesity markedly increases placental fatty acid transporter expression and fetal blood triglycerides at midgestation in the ewe. *Am J Physiol Regul Integr Comp Physiol.* (2010) 299:R1224–31. 10.1152/ajpregu.00309.2010 20844260 PMC2980459

[187] Davis J Mire E. Maternal obesity and developmental programming of neuropsychiatric disorders: an inflammatory hypothesis. *Brain Neurosci Adv.* (2021) 5:23982128211003484. 10.1177/23982128211003484 33889757 PMC8040564

[188] Tavian M Robin C Coulombel L Péault B. Thehuman embryo, but not its yolk sac, generates lympho-myeloid stem cells: mapping multipotent hematopoieticcell fate in intraembryonic mesoderm. *Immunity.* (2001) 15:487–95. 10.1016/s1074-7613(01)00193-5 11567638

[189] Farley AM Morris LX Vroegindeweij E Depreter ML Vaidya H Stenhouse FH Dynamics of thymus organogenesis and colonizationin early human development. *Development.* (2013) 140:2015–26. 10.1242/dev.087320 23571219 PMC3631974

[190] Emeny RT Gao D Lawrence DA. Beta1-adrenergic receptors on immune cells impair innate defenses against Listeria. *J Immunol.* (2007) 178:4876–84. 10.4049/jimmunol.178.8.4876 17404268

[191] Zieziulewicz TJ Mondal TK Gao D Lawrence DA. Stress-induced effects, which inhibit host defenses, alter leukocyte trafficking. *Cell Stress Chaperones.* (2013) 18:279–91. 10.1007/s12192-012-0380-0 23111563 PMC3631090

